# Insights into the role of deep-sea squids of the genus *Histioteuthis* (Histioteuthidae) in the life cycle of ascaridoid parasites in the Central Mediterranean Sea waters

**DOI:** 10.1038/s41598-021-86248-5

**Published:** 2021-03-30

**Authors:** Marialetizia Palomba, Simonetta Mattiucci, Fabio Crocetta, David Osca, Mario Santoro

**Affiliations:** 1 Department of Integrative Marine Ecology, Stazione Zoologica Anton Dohrn, Villa Comunale, 80121 Naples, Italy; 2 Department of Public Health and Infectious Diseases, Section of Parasitology, Sapienza University of Rome, 00185 Rome, Italy

**Keywords:** Ecology, Molecular biology

## Abstract

Ascaridoid nematodes comprise a wide range of heteroxenous parasites infecting top fish predators and marine mammals as definitive hosts, with crustaceans, squids, and fishes acting as intermediate/paratenic hosts. Limited data exist on the species and role of several intermediate and paratenic hosts in the life cycle of these parasites. In the aim of adding knowledge on the role of squid species in their life cycle, we have here investigated the larval ascaridoid nematodes collected from the deep-sea umbrella squid *Histioteuthis bonnelli* and the reverse jewel squid *Histioteuthis reversa* captured in the Central Mediterranean Sea (Tyrrhenian Sea). Morphological study and sequence analysis of the internal transcribed spacer (ITS) regions of the ribosomal DNA (rDNA) and the mitochondrial cytochrome *c* oxidase subunit 2 (mtDNA *cox*2) gene locus revealed the occurrence of *Anisakis physeteris* and of an unidentified species of the genus *Lappetascaris*. Sequence analysis revealed that specimens of *Lappetascaris* from both squid species matched at 100% sequences previously deposited in GenBank from larval ascaridoids collected in octopuses of the genus *Eledone* of the Mediterranean Sea. The Bayesian inference tree topology obtained from the analysis of the fragments amplified showed that *Lappetascaris* specimens were included in a major clade comprising *Hysterothylacium* species collected in fishes of the families Xiphiidae and Istiophoridae. As regards the site of infection in the squid host species, *A. physeteris* larvae predominated (60.7%) in the gonads, while those of *Lappetascaris* (76.3%) were found infecting the mantle musculature. The overall high values of parasitic load suggest both squid species as transmitting hosts of third stage larvae of *Lappetascaris* to top predator fishes, as well as the umbrella squid as an intermediate/paratenic host in the life cycle of *A. physeteris* in the Mediterranean Sea.

## Introduction

Histioteuthidae Verrill, 1881 is a family of pelagic cephalopods distributed circumglobally in the midwaters of the oceans, from the subarctic to the subantarctic regions^1^. The umbrella squid *Histioteuthis bonnellii* (Férussac, 1835) and the reverse jewel squid *Histioteuthis reversa* (Verrill, 1880) are the only species inhabiting the Mediterranean Sea^2^, where they are usually found between 500 and 1500 m depth^1,3–6^. Both species are important prey-resources for higher trophic levels, such as those constituted by marine mammals and top fish predators; they are also voracious consumers of crustaceans, other cephalopods, and fishes^1,6–12^.

Host-species interaction through food webs allows transmission and maintenance of biological cycles of most of the parasites in marine ecosystems^13,14^. Parasites constitute an important component of every marine community showing a high diversity in their life-cycles^15–17^. Cephalopods, as intermediate or paratenic hosts in the life cycle of heteroxenous parasites, can accumulate them throughout their lifespan, thus increasing the chance of predation by the next host and, consequently, the probability of parasite transmission. This is especially relevant for ascaridoid nematodes, which use squids as intermediate and/or paratenic hosts^14,18,19^ and marine mammals or teleostean fishes as definitive ones^20^.

Likely due to their elusiveness, most of the records so far available in the literature about the umbrella and reverse jewel squids from the Mediterranean Sea have been limited to occasional captures with the description of morphometric features or data derived from the gastric contents of teuthophagous predators^21–23^, or because they were included within studies on the cephalopod faunas of certain geographic areas^6,24,25^. Moreover, the only published data regarding ascaridoid nematodes in the Mediterranean histioteuthids is a morphological study by Culurgioni et al.^26^, reporting low prevalence and abundance levels of larval forms of *Lappetascaris* sp. Type A in the umbrella squid and the reverse jewel squid, as well as the occurrence of third stage larvae of *Anisakis* sp*.* morphotype II (sensu Berland, 1961) in the umbrella squid from the Sardinian Channel (Western Mediterranean).

In the present paper, a genetic/molecular approach was applied to identify, at the lower possible taxonomic level, larval ascaridoid nematodes collected from poorly known squids species (i.e., the umbrella squid and reverse jewel squid) from the Tyrrhenian Sea in order to: *i)* add knowledge on the role of these squids species in nematode parasites having in top predators of a marine food webs their definitive hosts; *ii)* provide data on their infection level and site of infection in the hosts. Identification of the examined squid hosts was also included by means of genetic/molecular analysis.

## Materials and methods

### Sampling

A total of 10 specimens of the genus *Histioteuthis* d'Orbigny [in Férussac & d'Orbigny], 1841 were collected from off Campania coast (Tyrrhenian Sea, Mediterranean), during July and August 2020. Nine specimens were obtained from off Ischia Island (Gulf of Naples) (~ 40°35′30″ N, 14°00′37″ E) and a single individual from the Gulf of Salerno (~ 40°34′30″ N, 14°40′00″ E). In particular, they constituted the by-catch of commercial and scientific trawling operations (red shrimp’s fishery) held with commercial fishing vessels equipped with bottom trawl nets (mouth of 3 × 4 m in height and width, respectively; 40 mm mesh size), towed at ~ 2–2.5 kn on muddy bottoms at ~ 450–600 m depth see^27^. Procedures for this study were performed in accordance with the permit n. 0008453 (issued May 15, 2020) by the Italian Ministry of Agricultural, Food and Forestry Policies, guide for the care and use of animals by the Italian Ministry of Health and the ARRIVE guidelines.

### Morphological and molecular identification of the squids

After the sampling, squids were transferred, in iceboxes, to the laboratory, where the specimens were identified to the species level according to their morphological characters^3,5,28^. Subsequently, they were weighed (Wt) to the nearest 0.1 g and measured (dorsal mantle length, DML) to the nearest 0.1 cm. Sex was determined before the parasitological inspection by gonadal examination. The identification to the species level was then supported by direct sequencing of PCR products for the barcode gene locus.

Total genomic DNA was extracted from squid muscle samples using the DNeasy® Blood & Tissue kit (QIAGEN), following the manufacturer’s protocol. A partial sequence of the mitochondrial cytochrome *c* oxidase subunit 1 gene locus (mtDNA *cox*1) was amplified from each specimen using both the primers developed by Folmer et al.^29^ [LCO-1490 (forward) 5′-GGTCAACAAATCATAAAGATATTGG-3′; HCO-2198 (reverse) 5′-TAAACTTCAGGGTGACCAAAAATCA-3′] and their degenerated version by Meyer^30^ [dgLCO-1490 (forward) 5′-GGTCAACAAATCATAAAGAYATYGG-3′; dgHCO-2198 (reverse) 5′-TAAACTTCAGGGTGACCAAARAAYCA-3′]. The polymerase chain reactions (PCRs) were conducted in 25 *μ*L volume reaction, containing 2.5 *μ*L of Roche buffer (10 ×), 2.5 *μ*L (2 mM) of dNTPack Mixture (Roche), 1 *μ*L of each forward and reverse primers (10 *µ*M), 0.25 *μ*L (5 U/*μ*L) of Roche Taq DNA polymerase, 1 *μ*L of DNA (15 ng/*μ*L) and sterilized distilled water up to 25 *μ*L. Amplifications were performed with the following conditions: initial denaturation at 95 °C (5 min), followed by 39 cycles of denaturation at 95 °C (1 min), annealing at 45 °C (1 min), extension at 72 °C (1 min), with a final extension at 72 °C (5 min). The successful PCR products were purified, and Sanger sequenced through an Automated Capillary Electrophoresis Sequencer 3730 DNA Analyzer (Applied Biosystems), using the BigDye® Terminator v3.1 Cycle Sequencing Kit (Life Technologies). Forward and reverse sequences obtained were assembled using Sequencher v. 5.0.1 (GeneCodes Co.) and compared with reference sequences using BLASTn^31^.

### Parasitological analysis of the squids

For the parasitological examination, each squid specimen was cut along the ventral mid-line of the mantle, the organs were removed, placed individually in plastic Petri dishes (200 mm in diameter), opened, and studied for metazoan parasites under a dissecting microscope. The mantle of each specimen was dissected in small pieces (1 cm × 1 cm) and examined under the dissecting microscope. Parasites found embedded in the mantle tissue were extracted using scissors and tweezers. All the removed nematodes were subsequently counted, washed in physiological saline solution, and preserved in 70% ethanol or frozen at − 20 °C for morphological and molecular identification, respectively. Larval nematodes were studied and photographed using a dissecting microscope and a compound microscope both equipped with ZEN 3.1 imaging system (Zeiss). They were morphologically assigned to the genus level according to the morphological features^32–34^. Descriptors of the parasite distribution used in the present study follow Bush et al.^35^.

### Molecular identification of ascaridoid parasites

Total genomic DNA from ∼2 mg of each parasite was extracted using Quick-gDNA Miniprep Kit (ZYMO RESEARCH) following the standard manufacturer-recommended protocol. The ITS region of rDNA including the first internal transcribed spacer (ITS-1), the 5.8S gene, the second transcribed spacer (ITS-2), and ∼70 nucleotides of the 28S gene, was amplified using the primers NC5 (forward; 5′-GTAGGTGAACCTGCGGAAGGATCATT-3′) and NC2 (reverse; 5′-TTAGTTTCTTTTCCTCCGCT-3′)^36^. PCRs were carried out in a 15 *µ*L volume containing 0.3 *µ*L of each primer 10 mM, 2.5 *µ*L of MgCl_2_ 25 mM (Promega), 15 *µ*L of 5 × buffer (Promega), 0.3 *µ*L of DMSO, 0.3 *µ*L of dNTPs 10 mM (Promega), 0.3 *µ*L (5 U/*μ*L) of Go-*Taq* Polymerase (Promega) and 2 *µ*L of total DNA. PCR temperature conditions were the following: 94 °C for 5 min (initial denaturation), followed by 30 cycles at 94 °C for 30 s (denaturation), 55 °C for 30 s (annealing), 72 °C for 30 s (extension) and followed by post-amplification at 72 °C for 5 min. Additionally, the mtDNA *cox*2 locus was sequenced in a subsample of 10 larvae of both the genera *Anisakis* Dujardin, 1845 and *Lappetascaris* Rasheed, 1965, randomly selected among those sequenced at the ITS region of rDNA, using the primers 211F (5′-TTTTCTAGTTATATAGATTGRTTYAT-3′) and 210R (5′-CAC CAACTCTTAAAATTATC-3′)^37,38^. PCRs were carried out in a 25 *µ*L volume containing 2 *µ*L of each primer 10 mM, 4 *µ*L of MgCl_2_ 25 mM (Promega), 5 *µ*L of 5 × buffer (Promega), 2 *µ*L of dNTPs 10 mM (Promega), 0.25 *µ*L (5 U/*μ*L) of Go-*Taq* Polymerase (Promega) and 3 *µ*L of total DNA. The amplification protocol was performed using the following conditions: 94 °C for 3 min (initial denaturation), followed by 35 cycles at 94 °C for 30 s (denaturation), at 46 °C for 1 min (annealing), at 72 °C for 90 s (extension), and a final extension at 72 °C for 10 min.

The successful PCR products were purified, and Sanger sequenced through an Automated Capillary Electrophoresis Sequencer 3730 DNA Analyzer (Applied Biosystems), using the BigDye® Terminator v3.1 Cycle Sequencing Kit (Life Technologies).

The obtained sequences were analysed, edited, and assembled by Sequence Matrix v. 1.7.8^39^ and compared with those available in GenBank using BLASTn^31^ (see Tables 1, 2). JModelTest v. 2.1.10^40,41^ was used to select the best fit model using the Akaike Information Criterion (AIC)^42–44^. Phylogenetic trees of the ITS-1 and ITS-2 region and *cox*2 gene locus were constructed using Bayesian inference (BI) with MrBayes, v. 3.2.7^45^. The Bayesian posterior probability analysis was performed using the MCMC algorithm, with four chains, 0.2 as the temperature of heated chains, 1,000,000 generations, with a subsampling frequency of 100 and a burn-in fraction of 0.25. Posterior probabilities were estimated and used to assess support for each branch. Values with a 0.90 posterior probability were considered well-supported. Genetic distances were computed using the Kimura 2-Parameters (K2P) model^46^ with 1000 bootstrap re-samplings, using MEGA Software, version 7.0^47^.

**Table 1:** Species, stage (A: adult, L4: fourth larval stage, L3: third larval stage), host, geographical location, and accession number of sequences of ITS rDNA of *Hysterothylacium* species included in the Bayesian inference shown in Fig. 2.

Species	Stage	Host	Geographical location	Accession number	References
*H. aduncum* (Rudolphi, 1802)	A	*Melanogrammus aeglefinus*	Northeast Atlantic Ocean	MW131976	^76^
*H. amoyense* (Hsü, 1933) Deardorff & Overstreet, 1980	L3	*Lophius litulon*	Chinese waters	MH211527	^77^
*H. auctum* (Rudolphi, 1802) Deardorff & Overstreet, 1981	L3	*Zoarces viviparus*	Baltic Sea	AF115571	^78^
*H. australe* Shamsi, 2016	A	*Seriola lalandi*	Australian waters	HE862216-HE862225	^63^
*H. bidentatum* (Linstow, 1899) Deardorff & Overstreet, 1981	–	–	–	AY603539	GenBank unpublished
*H. brucei* Shamsi, 2016	A	*Kajikia audax*	Australian waters	HE862222-HE862230	^63^
*H. deardoffoverstreetorum* Knoff, Felizardo, Iñiguez, Maldonado, Torres, Magalhães Pinto & Gomes, 2012	–	*Cynoscion nebulosu*	South Carolina coast	MF668866	GenBank unpublished
*H. fabri* (Rudolphi, 1819) Deardorff & Overstreet, 1980	L4	*Lophius litulon*	Chinese waters	MH211492	^77^
*H. fortalezae* (Klein, 1973) Deardorff & Overstreet, 1981	L3	*Maurolicus weitzmani*	Gulf of Mexico	KX098563	^79^
*H. liparis* Li, Xu & Zhang, 2007	L4	*Lophius litulon*	Chinese waters	MH211547	^77^
*H. longilabrum* Li, Liu & Zhang, 2012	A	*Siganus* sp.	Chinese waters	JQ520159	^80^
*H. persicum* Shamsi, Ghadam, Suthar, Mousavi, Soltani & Mirzargar, 2016	A	*Scomberomorus commerson*	Persian Gulf	LT576367-LT576370	^81^
*H. reliquiens* (Norris & Overstreet, 1975) Deardorff & Overstreet, 1981	A	*Brachirus orientalis*	Persian Gulf	MF061682	^82^
*H. rigidum* (Rudolphi, 1809) Deardorff & Overstreet, 1980	–	*Lophius piscatorius*	Ireland, Porcupine Bank	HF680323	GenBank unpublished
*H. sinense* Li, An & Zhang, 2007	L3	*Conger myriaster*	Chinese waters	MF539804	^82^
*H. tetrapteri* (Bruce & Cannon, 1989) Moravec & Justine, 2005	–	–	Chinese waters	KF601901	GenBank unpublished
*H. thalassini* Bruce, 1990	A	*Priacanthus macracanthus*	Chinese waters	JX982129	^83^
*H. kajikiae* Shamsi, 2016	A	*Kajikia audax*	New Caledonia	HE862220-HE862229	^63^
*H. zhoushanense* Li, Liu & Zhang, 2012	L3	*Lophius litulon*	Chinese waters	MH211555	^77^
*H.* larval type III	L3	*Lutjanus* sp.	Queensland waters (Australia)	FN811721-FN811678	^84^
*H.* larval type IV	L3	*Halieutaea stellata*	Chinese waters	KP203840	^85^
*H.* larval type IV-A	L3	*Apogonichthyoides taeniatus*	Chinese waters	KP326500	^86^
*H.* larval type IV-B	L3	*Sardionops sagax*	Australian waters	MK161418-MK161443	^87^
*H*. larval type IV-C	L3	*Sillago flindersi*	Australian waters	JN631798-JN631805	^88^
*H.* larval type IV-D	L3	*Sillago flindersi*	Australian waters	JN631799-JN631806	^88^
*H.* larval type V	L3	*Lutjanus carponotatus*	Queensland waters	FN811738-FN811699	^84^
*H.* larval type VI	L3	*Chaetodon lineolatu*	Queensland waters	FN811740-FN811701	^84^
*H.* larval type VII	L3	*Caesio cunning*	Queensland waters	FN811749-FN811709	^84^
*H.* larval type VIII	L3	*Engraulis australis*	Australian waters	MK161423-MK161448	GenBank unpublished
*H.* larval type X	L3	*Upeneichthys lineatus*	Australian waters	KC437340-KC437350	^89^
*H.* larval type XI	L4	*Seriola lalandi*	Australian waters	FN811763-FN811717	^84^
*H.* larval type XII	L4	*Lutjanus carponotatus*	Queensland waters	FN811767-FN811720	^84^
*H.* larval type XIV	L3	*Engraulis australis*	Australian waters	MK161424-MK161449	^87^
*H.* larval type XV	L3	*Otolithes ruber*	Persian Gulf, Iran	LT576354-LT576363	^81^
*H.* larval type XVII	L3	*–*	Queensland waters	MG594313-MG594336	^90^
*H.* larval type XVIII	L3	*Engraulis australis*	Australian waters	MK161426-MK161451	^87^
*Ascaris lumbricoides* Linnaeus, 1758	A	*Homo sapiens*	Japan	AB571298	^91^

(–: data not stated).

**Table 2:** Species, stage (A: adult, L4: fourth larval stage, L3: third larval stage), host, geographical location, and accession number of sequences of mtDNA *cox*2 of *Hysterothylacium* species included in the Bayesian inference shown in Fig. 3.

Species	Stage	Host	Geographical location	Accession number	References
*H. aduncum* (Rudolphi, 1802)	–	*Theragra chalcogramma*	South Korea	KY270874	GenBank unpublished
*H. amoyense* (Hsü, 1933) Deardorff & Overstreet, 1980	A	*Muraenesox cinereus*	Chinese waters	MF120253	^64^
*H. corrugatum* Deardorff & Overstreet, 1981	A	*Xiphias gladius*	Mediterranean Sea	MW456072	^92^
*H. deardoffoverstreetorum* Knoff, Felizardo, Iñiguez, Maldonado, Torres, Magalhães Pinto & Gomes, 2012	L3	*Mullus argentinae*	Southeast coast of Brazil	MF189875	^93^
*H. fabri* (Rudolphi, 1819) Deardorff & Overstreet, 1980	L4	*Zeus faber*	Turkey Mediterranean coast	KC862609	^94^
*H. fortalezae* (Klein, 1973) Deardorff & Overstreet, 1981	–	–	–	AF179914	^95^
*H. liparis* Li, Xu & Zhang, 2007	A	*Liparis tanakae*	Chinese waters	MF120251	^64^
*H. longilabrum* Li, Liu & Zhang, 2012	A	*Siganus fuscescens*	Chinese waters	MF120247	^64^
*H. reliquiens* (Norris & Overstreet, 1975) Deardorff & Overstreet, 1981	A	*Brachirus orientalis*	Arabian Gulf	KX825845	^95^
*H. sinense* Li, An & Zhang, 2007	A	*Conger myriaster*	Chinese waters	MF120254	^64^
*H. tetrapteri* (Bruce & Cannon, 1989) Moravec & Justine, 2005	A	*Kajikia audax*	Chinese waters	MF120256	^64^
*H. thalassini* Bruce, 1990		*Priacanthus macracanthus*	Chinese waters	MF120250	^64^
*H. zhoushanense* Li, Liu & Zhang, 2012	A	*Pseudorhombus oligodon*	Chinese waters	MF120248	^64^
*Ascaris lumbricoides* Linnaeus, 1758	A	*Homo sapiens*	Denmark, Odense	KY368760	GenBank unpublished
*Toxocara canis* Werner, 1782	–	–	–	AF179923	^95^

(–: data not stated).

## Results

### Identification of squid species

Based on their external morphology (mantle length, development of the inner web, and buccal membrane), the *Histioteuthis* specimens were identified as belonging to the umbrella squid (8 specimens, of which 7 from the Gulf of Naples and 1 from the Gulf of Salerno) and the reverse jewel squid (2 specimens from the Gulf of Naples). All squids were males showing Wt and DML ranges as it follows 930–2450 g and 111–177 mm for the umbrella squids, and 140–206 g and 86–95 mm for the reverse jewel squids, respectively.

Partial sequences of the mtDNA *cox*1 were obtained from all the specimens analysed here [8 umbrella squids (691 bp) and 2 reverse jewel squids (693 bp)]. The sequences of umbrella squids here obtained from the Mediterranean Sea showed > 99.5% of identity with sequences already deposited for the same species from the Atlantic Ocean^48,49^, whereas those from the reverse jewel squid showed 98.45–100% of identity with those of the same species from the Atlantic Ocean^48,49^, thus confirming their identity as achieved also by morphological analysis.

### Parasitological general data

**Figure 1:**
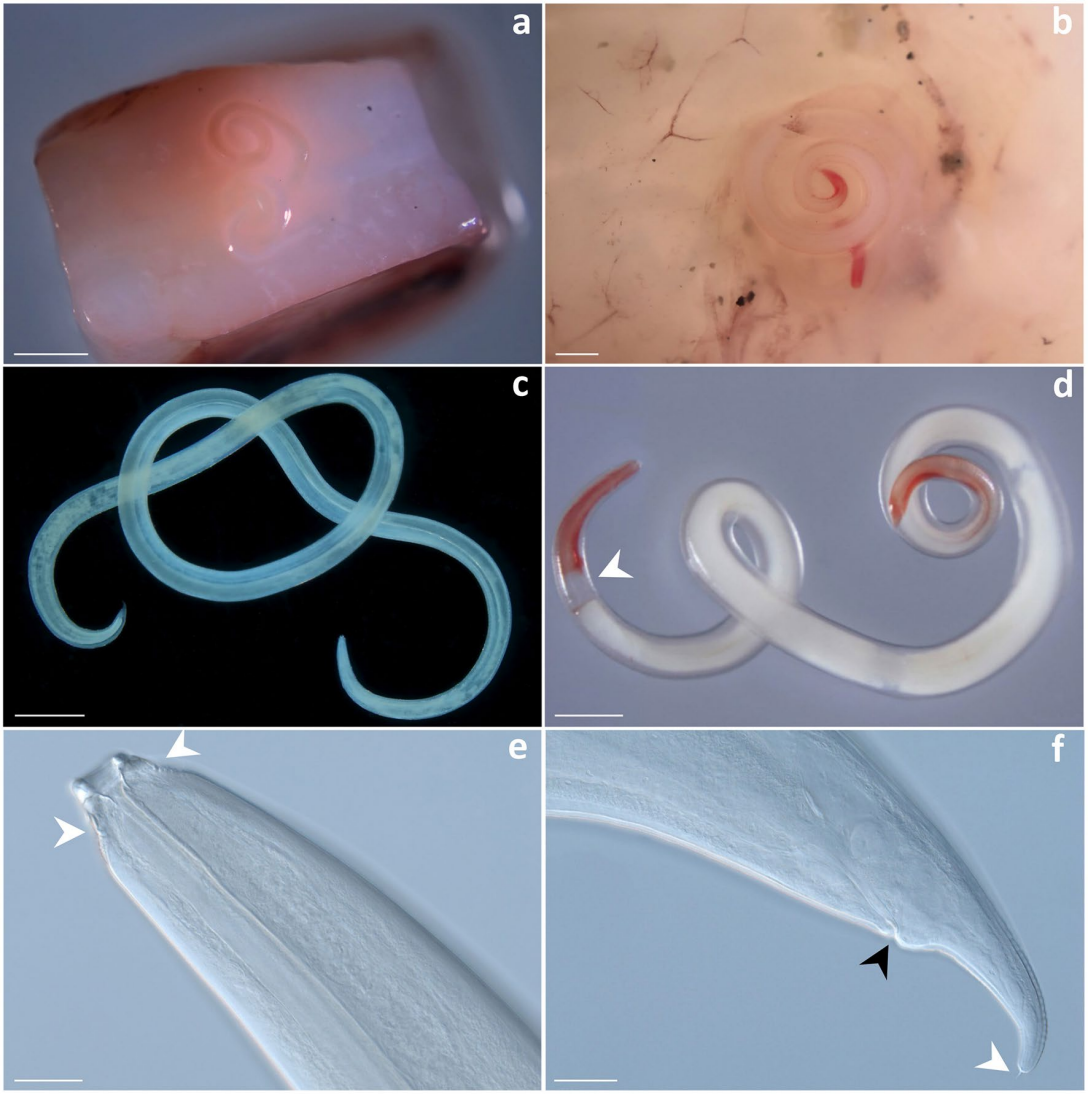
*Lappetascaris* sp. and *Anisakis physeteris* in *Histioteuthis* squids collected from the Tyrrhenian Sea. *Lappetascaris* larva in the mantle musculature of an umbrella squid (**a**). *Anisakis physeteris* in the testis of an umbrella squid. Note the worm extremities reddish in colour (**b**). Microscopic view of *Lappetascaris* sp. larva (**c**) and *A. physeteris* showing the extremities reddish in colour and ventriculus (arrow) (**d**). Cephalic extremity of *Lappetascaris* sp. larva showing sclerotized formations (arrows) (**e**). Caudal extremity of *Lappetascaris* sp. larva showing anus (black arrow) and cuticular spike (white arrow) (**f**). Scale bar: 2000 µm (**a**); 1000 µm (**b**,**c**,**d**); 50 µm (**e**,**f**).

An overall of 161 ascaridoid nematode larvae was collected from the two squid species. Out of them, 133 (82.6%) were morphologically assigned to the genus *Lappetascaris* showing morphological features of the third stage larvae (L3) Type A (Fig. 1a,c,e,f). The following measurements were achieved on 10 larvae. They were*:* 26.05 ± 3.16 mm in body length (range: 20.60–29.27 mm) and 0.45 ± 0.10 mm in body width (range: 0.32–0.63 mm), and whitish in colour (Fig. 1a,c). In addition, a total of 28 (17.4%) nematode larvae were morphologically assigned to the genus *Anisakis* showing features of the L3 Type II larvae (sensu Berland, 1961) (Fig. 1b,d). The measurements obtained on 10 larvae were 26.24 ± 1.94 mm in body length (range: 21.40–28.35 mm) and 0.63 ± 0.01 mm in body width (range: 0.62–0.75 mm), with both extremities reddish in colour (Fig. 1b,d).

### Molecular/genetic analysis of the ascaridoid nematodes

According to the obtained sequences (850 bp) at the ITS region of the rDNA, 28 *Anisakis* sp. Type II larvae showed 100% identity with the sequences of *A. physeteris* (Baylis, 1923) previously deposited in GenBank (accession numbers MF668924–MF668926). The mtDNA *cox*2 gene locus (580 bp), sequenced in a subsample of 10 larvae, also identified the larvae as *A. physeteris*. Those sequences matched at 99–100% with the mtDNA *cox*2 sequences of *A. physeteris* obtained in previous works and deposited in GenBank (accession number KY595212). Sequences of the species *A. physeteris* here obtained were deposited in GenBank with the accession numbers MW697752-53 (ITS region of the rDNA) and MW691145-46 (mtDNA *cox*2).

The sequences (850 bp) of the ITS region of rDNA obtained from the 133 *Lappetascaris* Type A larvae showed 100% identity with the sequences of larvae morphologically indicated as *Lappetascaris* sp. from octopuses of the genus *Eledone* Leach, 1817 sequenced by Guardone et al.^50^ and deposited by the same authors in GenBank as *Hysterothylacium* sp. (accession numbers MT365530–37). The BLAST analysis of the sequences at mtDNA *cox*2 gene locus (580 bp) obtained from 10 *Lappetascaris* sp. larvae showed 88–89% similarity with *H. corrugatum* Deardorff & Overstreet, 1981 (accession number MW456072).

**Figure 2:**
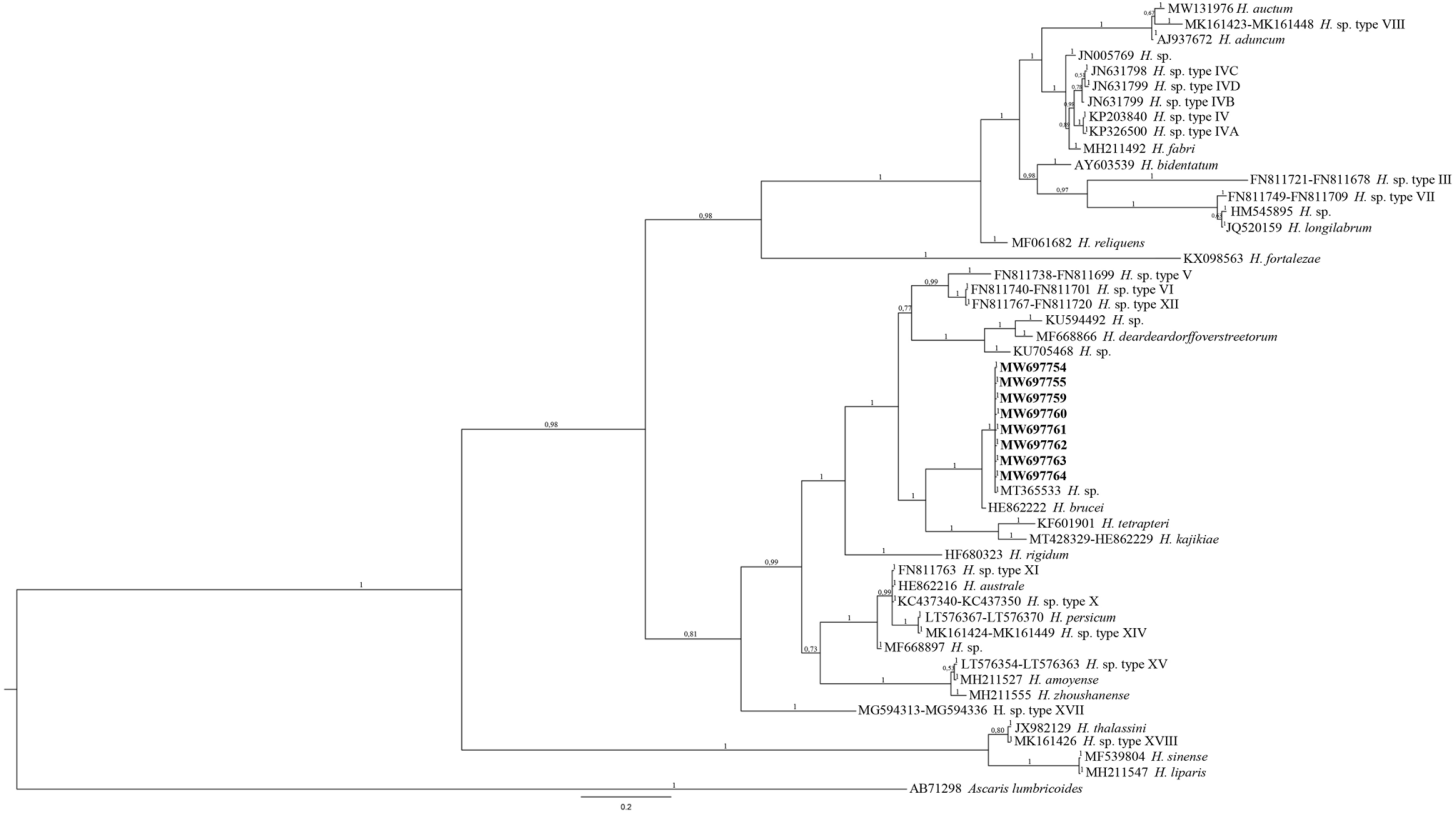
Phylogenetic concatenated tree from Bayesian inference based on ITS-1 and ITS-2 sequences of *Lappetascaris* sp. obtained in the present study, with respect to the sequences of raphidascaridid species at the same gene loci available in GenBank. The analysis was performed by MrBayes, v. 3.2.7, using the GTR + G substitution model, as implemented in jModeltest 2.1.10. *Ascaris lumbricoides* was used as outgroup. Sequences obtained in the present study are in bold. Tree was drawn using FigTree v. 1.3.1 (http://tree.bio.ed.ac.uk/software/figtree/).

The BI tree topology as inferred from the phylogenetic analysis of the sequences obtained at the ITS region of rDNA of *Lappetascaris* larvae showed that they are all clustering in the same clade, supported with high probability value, which also includes the sequences MT365530–37 deposited by Guardone et al.^50^ (Fig. 2). The sequences included in this clade showed a close relationship with the sequence of *H. brucei* Shamsi, 2016 retrievable in GenBank (Fig. 2). Indeed, the distance values resulted to be K2P = 0.007 and K2P = 0.11, respectively at the ITS-1 and ITS-2 loci, between *Lappetascaris* sp. and *H. brucei*. The sequences of *Lappetascaris* sp. showed a higher K2P value with respect to the species *H. deardoffoverstreetorum* Knoff, Felizardo, Iniguez, Maldonado, Torres, Magalhaes Pinto & Gomes, 2012 (K2P = 0.06 at ITS-1, K2P = 0.18 at the ITS-2). On the other hand, the BI tree also showed that the clade comprising *Lappetascaris* sp. and some species of *Hysterothylacium* here considered and available in GenBank [i.e., *H. brucei*, *H. tetrapteri* (Bruce & Cannon, 1989) Moravec & Justine, 2005, *H. deardoffoverstreetorum*], is well-supported with high value of probability, well distinct from the other clades (Fig. 2) including other species of the genus *Hysterothylacium*. Indeed, the highest values of K2P between *Lappetascaris* sp. and other *Hysterothylacium* species were found with respect to *H. fortalezae* (Klein, 1973) Deardorff & Overstreet, 1981 (K2P = 0.21 at the ITS-1, KP2 = 0.59 at the ITS-2) (Fig. 2).

**Figure 3:**
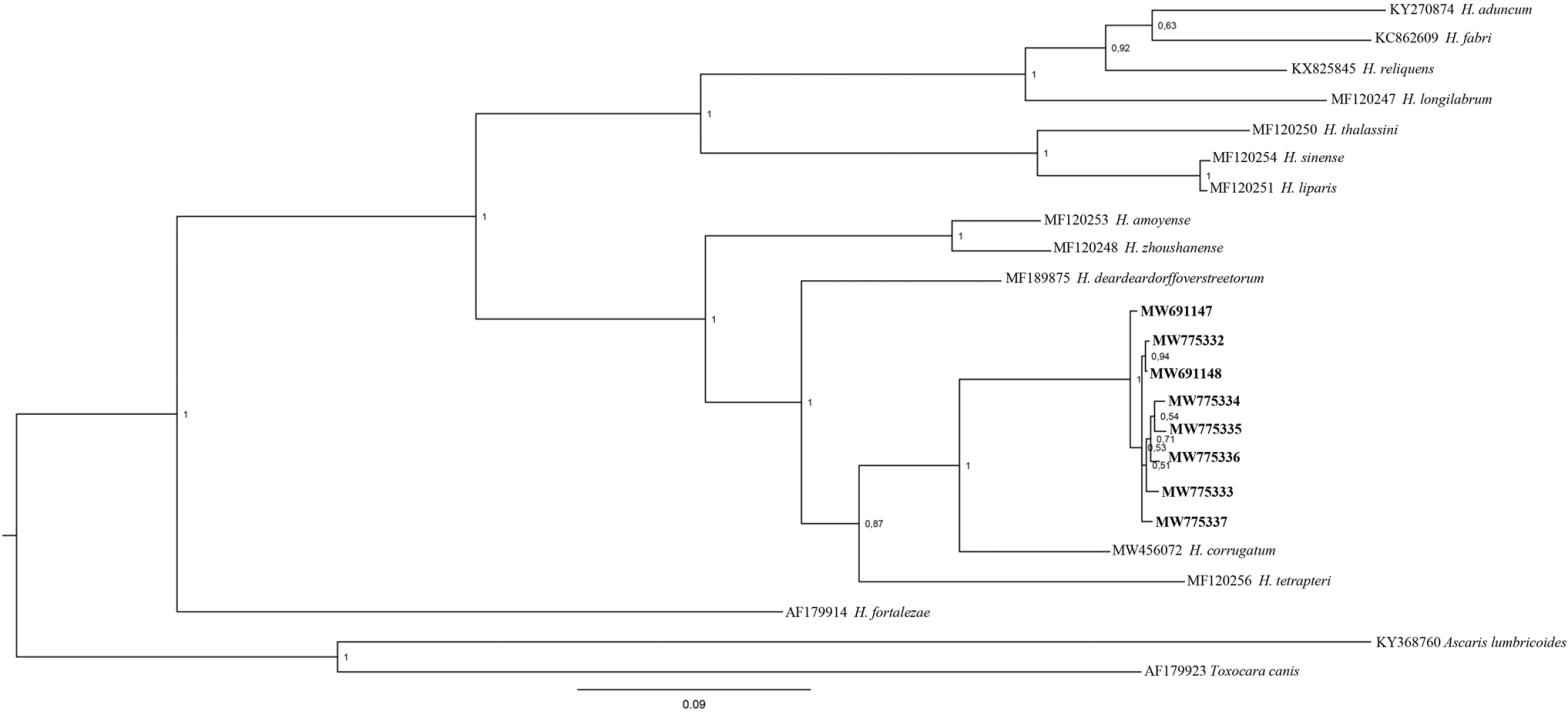
Phylogenetic tree from Bayesian inference on *cox*2 sequences of *Lappetascaris* sp. obtained in the present study, with respect to the sequences of raphidascaridid species at the same gene loci available in GenBank. The analysis was performed by MrBayes, v. 3.2.7, using the TrN + I + G substitution model, as implemented in jModeltest 2.1.10. *Ascaris lumbricoides* and *Toxocara canis* were used as outgroup. Sequences obtained in the present study are in bold. Tree was drawn using FigTree v. 1.3.1 (http://tree.bio.ed.ac.uk/software/figtree/).

Similar tree topology was shown by the BI inference using the sequence’s analysis of the mtDNA *cox*2 gene locus. In particular, the sequences of *Lappetascaris* sp. clustered in a well-supported distinct phylogenetic lineage (Fig. 3) with other species of *Hysterothylacium,* such as *H. deardoffoverstreetorum*, *H. amoyense* (Hsu, 1933) Deardorff & Overstreet, 1980, *H. zhoushanense* Li, Liu & Zhang, 2012, *H. tetrapteri*, and *H. corrugatum*. The closer sequence similarity (K2P = 0.12 ± 0.016) was found between *Lappetascaris* and *H. corrugatum*. Higher level of K2P distance was found between the sequences of *Lappetascaris* and *H. fortalezae* (K2P = 0.26 ± 0.004) and with respect to *H. thalassini* Bruce, 1990 (K2P = 0.26 ± 0.006), which are, instead, included in the other well supported clade, with a high probability value (Fig. 3).

Sequences of *Lappetascaris* sp. obtained were deposited in GenBank with the accession numbers MW697754-55, MW750359-64 (ITS region) and MW691147-48, MW775332-37 (mtDNA *cox*2).

### Parasitic infection and site preferences

Squids were all found infected by at least 1 nematode larva. The maximum number of larvae was 32 and 11 in the umbrella squid and the reverse jewel squid, respectively. The umbrella squids were found to be infected by both nematode larval forms with prevalence of 87% and abundance (± standard deviation) of infection of 3.5 ± 3.2 for *A. physeteris*, and prevalence of 100% and abundance of 13.25 ± 9.2 for larvae of *Lappetascaris* respectively. The reverse jewel squids were infected only with *Lappetascaris* larvae with prevalence and abundance of 100% and 11 ± 0.0, respectively.

Regarding tissue distribution in the umbrella squid, out of the 28 larvae of *A. physeteris*, 17 (60.7%) were recorded in the testis (Fig. 1b), 6 (21.4%) were free in the body cavity, 2 (7.1%) were in the gills; the remaining larvae were respectively collected in the nidamental gland, the wall of the stomach, and the connective tissue surrounding the mantle muscle. Out of the 133 *Lappetascaris* larvae collected from both the umbrella and the reverse jewel squids, 100 (76.3%) were found in the mantle (Fig. 1a), 22 (16.5%) in the body cavity, 4 (3%) in the gills, 2 (1.5%) in the nidamental gland, and 1 (0.7%) was in the wall of the stomach.

## Discussion

Squids are considered the trophic bridge for many marine heteroxenous parasites, including the ascaridoid nematodes^14,51^. The heteroxenous biological cycle of marine ascaridoids is entirely embedded within the food web of marine ecosystems as it follows the trophic relationships among their hosts, as based on a prey-predatory system^52,53^. Adults of the family Raphidascarididae Hartwich, 1954 are common parasites of predatory teleosts and squids, while crustaceans and various species of fish act, respectively, as intermediate and paratenic hosts^33,54^. Definitive hosts of *Anisakis* nematodes of the family Anisakidae Railliet & Henry, 1912 are marine mammals (mainly cetaceans), while the intermediate and/or paratenic hosts are crustaceans, fishes, and squids^52,53^. Several parasitological available data on squids are based strictly on morphological studies. This does not allow the identification of most of the parasite larvae to the species level, which is of pivotal importance for understanding the host-parasite relationships. This is the case of larvae of anisakid nematodes previously found in the Mediterranean histioteuthids and morphologically assigned to *Anisakis* sp. Type II (sensu Berland, 1961).

In the present study, *Anisakis* Type II larvae detected in the umbrella squid were identified, by genetic/molecular markers, as *A. physeteris*, whose main definitive hosts are cetaceans of the family Physeteridae Gray, 1821^53,55^. This finding suggests the umbrella squid as a transport host in transmitting *A. physeteris* to Physeteridae in the Mediterranean Sea. Histioteuthid squids are numerically among the prey items most important for the sperm whale from different geographical areas^56,57^. This is also supported by the large amounts of beaks of umbrella squid and adult specimens of *A. physeteris* detected as co-occurring in the stomach of the sperm whales *Physeter macrocephalus* Linnaeus, 1758 and the pygmy sperm whale *Kogia breviceps* (de Blainville, 1838), definitive hosts of the parasite species in the Mediterranean basin, recently stranded along the Mediterranean coasts^53,55,58,59^.

Moreover, the occurrence of an unidentified species of the genus *Lappetascaris*, both in the umbrella and reverse jewel squids, was studied by morphological and genetic analysis. To date the genus *Lappetascaris* comprises three species: *L. lutjani* Rasheed, 1965, *L. suraiyae* Kalyankar, 1975, and *L. chandipurensis* Gupta & Masoodi, 1990, reported in a wide range of freshwater and brackish fishes from Pakistan, India, and Brazil^33,60,61^. Unfortunately, no sequences of *L. lutjani* (type species of the genus) are so far available in GenBank for comparison with the *Lappetascaris* larvae here sequenced. The morphology of the larvae of the present material was identical with that of *Lappetascaris* described by Nagasawa & Moravec^33^. These authors supposed that those larvae found in the mantle of the Japanese flying squid *Todarodes pacificus* (Steenstrup, 1880) from the western North Pacific Ocean would represent an undescribed species of *Lappetascaris* whose definitive host, according to the same authors, would be a yet unknown predatory marine fish; unfortunately, the same authors did not perform genetic/molecular analysis of those specimens. However, according to the tree topologies inferred from the BI analyses at both the nuclear and mitochondrial regions, it appears that species of genus *Lappetascaris* are phylogenetically closely related to other ascaridoid nematodes having in teleost fish of the family Xiphiidae Rafinesque, 1815 and Istiophoridae Rafinesque, 1815 their definitive hosts. Among them, there are the species *H. corrugatum* and *H. tetrapteri*; interestingly, these two species appear to be closely related in the BI tree, inferred from the mtDNA *cox*2, to the *Lappetascaris* larvae here studied (Fig. 3), and are parasites found at the adult stage in the swordfish *Xiphias gladius* Linnaeus, 1758 (i.e., *H. corrugatum*) and in the striped marlin *Kajikia audax* (Philippi, 1887) (i.e., *H. tetrapteri*)^62^. In addition, the BI tree inferred from the ITS region of rDNA sequences analysis showed that *Lappetascaris* larvae here sequenced are phylogenetically related to *H. brucei*, which also matures into fish species of the family Istiophoriidae, i.e., in the striped marlin^63^. Unfortunately, sequences at the mtDNA *cox*2 gene locus are not available for the species *H. brucei*; therefore, it was not possible to include them in the BI here obtained at that gene locus. Interestingly, the finding of a close phylogenetic relationship between *Lappetascaris* sp., *H. corrugatum*, *H. brucei*, and *H. tetrapteri* seems to suggest that the definitive host of the *Lappetascaris* specimens sequenced here would be a top predator teleost fish belonging to the family Xiphiidae and/or Istiophoriidae, whose species members are known to commonly prey on histioteutiid squids^9,10,21^.

Moreover, both the BI tree topologies (Figs. 2, 3) also show that all those raphidascaridid species here considered and *Lappetascaris* species are clustering in two major clades; they include raphidascaridid ascaridoid species maturing in teleost fishes, as also recently shown by a multilocus phylogenetic analysis of ascaridoid nematodes^64^. However, in the last study, species of genus *Lappetascaris* were not included. The phylogenetic and morphological analyses performed on the species so far included in the genus *Lappetascaris*, in comparison with as many as possible raphidascaridid species, as well as by using a multilocus genetic approach, will help to clarify the taxonomy of this group of marine nematodes.

Taking into account that a high parasitic load with *A. physeteris* larvae has been previously identified in the swordfish^65^, the finding of several *A. physeteris* larvae in the examined squid species seems to support that hypothesis. In addition, in our previous parasitological analysis, the swordfish was found to harbour, in its stomach lumen, several squid beaks of the species of genus *Histiotheuthis* as a residual part of their prey items^65^.

The supposed life-history strategy of this parasite might explain the finding of *A. physeteris* in the umbrella squid alone. It has been suggested that different species of *Anisakis* have evolved different life-history strategies occupying different ecological niches, also in terms of vertical distribution^53,64–67^. Indeed, each parasite species has its depth preferences, following the most common feeding ecology and depth range of its definitive host. In turn, the depth preferences determine the spectrum of paratenic and intermediate hosts^52,53,66,67^. For example, Mattiucci & Nascetti^52^, Klimpel et al.^70^ and Mattiucci et al.^53^ suggested a deeper water life cycle for the species *A. paggiae*, *A. physeteris*, *A. ziphidarum*, and *A*. *nascettii*, in contrast to an epipelagic life cycle for *A. pegreffii* and *A. simplex* (*s. s.*). The finding of third stage larvae of *A. simplex* (*s. s.*) in pelagic squid species corroborates that hypothesis^68,69^. While, only L3 larvae of *A. nascettii* were genetically identified in the deep greater hooked squid species *Moroteuthopsis ingens* (Smith, 1881)^71^. Both the umbrella and the reverse jewel squids here analyzed are opportunistic deep-sea predators; however, the umbrella squid usually reaches higher depths^1^. Thus, the present findings could be correlated to a different ecology, in terms of feeding behaviour, and/or to a different spatial and bathymetric distribution of the two histioteuthid species. However, the parasitological analysis carried out on a higher number of specimens of these squid species, as well as other deep squid species, would in future support this hypothesis.

In the Mediterranean Sea, the co-occurrence of *A. physeteris* and *Hysterothylacium* larvae was recently recorded in the southern shortfin squid *Illex coindetii* (Vérany, 1839), with prevalence ranging from 1 to 17%^72,73^. Unfortunately, the sequences derived from these studies are not available in GenBank for comparison. A total of 9 *Lappetascaris* larvae were also reported in 5 (6.7%) individuals of the curled octopus *Eledone cirrhosa* (Lamarck, 1798) and of the musky octopus *Eledone moschata* (Lamarck, 1798)^50^. In the present study, the overall prevalence of ascaridoid larvae found in the *Histioteuthis* squids was higher (100%) than that reported by Culurgioni et al.^26^ (from 1.83 to 4.5%). This difference could be addressed to some ecological drivers, such as geographical sampling area, prey availability, season or year of sampling, and size of the host as well as to the method of squid inspection. Likewise, the distribution and abundance of the definitive hosts have been suggested as pivotal factors capable to influence the prevalence of infection and parasite abundance^74,75^. For instance, definitive hosts of *A. physeteris* (i.e., mysticetes of the family Physeteridae and Kogiidae) release a large amount of parasite eggs into the seawater with their faeces, so host distribution largely determines where infection with this nematode occurs^74,75^. However, the reasons for the higher prevalence and abundance in the present study are impaired by the lack of data on the biological cycle of these nematodes and in general by missing data on the ecology and biology of *Histhioteuthis* squids in the Mediterranean basin.

In the present study, different preference for the site of infection were recorded for the two ascaridoid taxa. Larvae of *A. physeteris* were mainly found in the gonads (testes) (60.7%) of squids; in contrast, the *Lappetascaris* larvae were mainly found in the mantle musculature (76.3%). Different site preferences for larval forms of *Anisakis* spp. and *Lappetascaris* spp. are in accordance with previous studies. Localization of *Anisakis* larvae in gonads of squids with parasitic castration was the most important pathological change observed by Abollo et al.^14^, where nematodes caused the partial destruction and alteration of gonad tissue and partial inhibition of gamete formation in hosts. In contrast, the localization of *Lappetascaris* larvae in the mantle of both *Histioteuthis* squids agrees with Nagasawa & Moravec^33^, that found this site preference as the most common for the genus *Lappetascaris*.

The main limitation of this study can be considered the low number of squids examined which makes our results not definitive. However, due to the difficulty to obtain specimens of these species, and the scarce published data on both *Histioteuthis* squids and their ascaridoid nematodes from the Mediterranean Sea we believe this study provides ecological, molecular and phylogenetic data that allow for a better characterisation of these poorly known hosts and their parasites.

In conclusion, although further studies are still necessary to understand which is the source of infection of both parasite taxa that infected the present *Histioteuthis* squids, this study provided for the first time the molecular identification of ascaridoid nematodes found in the umbrella and reverse jewel squids, and highlight the importance of both squids as transmitting hosts of *Lappetascaris* larvae to still unknown top predator fishes, and of the umbrella squid as vector host of *A. physeteris* to Physeteridae cetaceans. Studies are currently under way to identify the definitive host for the present larval forms of *Lappetascaris*, according to the present data and known fishes which commonly feed on *Histioteuthis* squids in the Mediterranean basin.
